# TRENDS IN DEPRESSION AND ANXIETY AMONG OLDER ADULTS ACROSS COVID-19 PANDEMIC PHASES

**DOI:** 10.1093/geroni/igad104.1959

**Published:** 2023-12-21

**Authors:** Tiffany Hughes, Yueting Wang, Ruopu Song, Erin Jacobsen, Chung-Chou Chang, Mary Ganguli

**Affiliations:** University of Pittsburgh, Pittsburgh, Pennsylvania, United States; University of Pittsburgh, Pittsburgh, Pennsylvania, United States; Western Psychiatric Institute & Clinic, UPMC Presbyterian Shadyside, Pittsburgh, Pennsylvania, United States; University of Pittsburgh, Pittsburgh, Pennsylvania, United States; University of Pittsburgh, Pittsburgh, Pennsylvania, United States; University of Pittsburgh, Pittsburgh, Pennsylvania, United States

## Abstract

The effect of the COVID-19 pandemic on older adults’ mental health is especially salient given their increased vulnerability to the pathologic and social effects of both the disease and also the associated mitigation strategies. The purpose of this research is to examine trends in symptoms of depression and anxiety among older adults during two phases of the pandemic (pre- and post-vaccine in study region) compared to before the pandemic began. Data were from a longitudinal cohort study of adults aged 65 and older spanning 26 months prior to the pandemic (phase 1) through 17 months after the pandemic began (9 months pre-vaccine (phase 2) and 8 months post-vaccine (phase 3)). Interrupted time series analyses were used to test for differences in trends over time in depressive symptoms (log-mCES-D) and anxiety symptoms (log-GAD-7) during these phases. Trends in both depressive symptoms and anxiety significantly increased in phase 2 compared to phase 1, then significantly decreased in phase 3 compared to phase 2. The trend for depressive symptoms and anxiety in phase 3 was not significantly different than phase 1. These findings suggest that older adults’ mental health significantly worsened during the phase of the pandemic when a vaccine was unavailable, but returned to pre-pandemic estimates after a vaccine was available. Future studies are needed to unpack the contribution of vaccine availability and other factors to changes in mental health observed among older adults during the pandemic.

